# Live Births from Domestic Dog (*Canis familiaris*) Embryos Produced by *In Vitro* Fertilization

**DOI:** 10.1371/journal.pone.0143930

**Published:** 2015-12-09

**Authors:** Jennifer B. Nagashima, Skylar R. Sylvester, Jacquelyn L. Nelson, Soon Hon Cheong, Chinatsu Mukai, Colleen Lambo, James A. Flanders, Vicki N. Meyers-Wallen, Nucharin Songsasen, Alexander J. Travis

**Affiliations:** 1 Baker Institute for Animal Health, College of Veterinary Medicine, Cornell University, Ithaca, New York, United States of America; 2 Atkinson Center for a Sustainable Future, Cornell University, Ithaca, New York, United States of America; 3 Smithsonian Conservation Biology Institute, National Zoological Park, Front Royal, Virginia, United States of America; 4 Department of Clinical Sciences, College of Veterinary Medicine, Cornell University, Ithaca, New York, United States of America; Michigan State University, UNITED STATES

## Abstract

Development of assisted reproductive technologies (ART) in the dog has resisted progress for decades, due to their unique reproductive physiology. This lack of progress is remarkable given the critical role ART could play in conserving endangered canid species or eradicating heritable disease through gene-editing technologies—an approach that would also advance the dog as a biomedical model. Over 350 heritable disorders/traits in dogs are homologous with human conditions, almost twice the number of any other species. Here we report the first live births from *in vitro* fertilized embryos in the dog. Adding to the practical significance, these embryos had also been cryopreserved. Changes in handling of both gametes enabled this progress. The medium previously used to capacitate sperm excluded magnesium because it delayed spontaneous acrosome exocytosis. We found that magnesium significantly enhanced sperm hyperactivation and ability to undergo physiologically-induced acrosome exocytosis, two functions essential to fertilize an egg. Unlike other mammals, dogs ovulate a primary oocyte, which reaches metaphase II on Days 4–5 after the luteinizing hormone (LH) surge. We found that only on Day 6 are oocytes consistently able to be fertilized. *In vitro* fertilization of Day 6 oocytes with sperm capacitated in medium supplemented with magnesium resulted in high rates of embryo development (78.8%, n = 146). Intra-oviductal transfer of nineteen cryopreserved, *in vitro* fertilization (IVF)-derived embryos resulted in seven live, healthy puppies. Development of IVF enables modern genetic approaches to be applied more efficiently in dogs, and for gamete rescue to conserve endangered canid species.

## Introduction

There is great and growing interest in better utilizing the domestic dog as a biomedical research model. Domestic dogs exhibit spontaneous occurrence of cancers, and as pets, they are exposed to environmental factors common to humans [1, 2]. The 350 traits/disorders identified in the dog with potential to be models for human disease are almost twice that of any other species [3]. In addition to improving understanding of the genetic basis of disease, these studies will also enable removal of deleterious traits from breeds, a significant advancement in animal health and welfare. To realize this scientific and clinical potential, we must be able to precisely control canine reproduction and enable access to the germline. The absence of true canine embryonic stem cells capable of germline transmission has left somatic cell nuclear transfer as the best approach both to transgenesis [4, 5] and to propagate genetically valuable working dogs [6]. New technologies such as CRISPR [7], should provide significant advantages, and could be enhanced considerably by use of ART such as IVF. Other applications, including genome rescue in endangered canid species, also require these technologies. Currently, when genetically valuable individuals die, we collect and freeze ovarian tissue and sperm. Yet these tissues and cells are useless if they cannot be used to produce offspring. Despite decades of attempts at IVF, no live births have previously been reported [8].

Failure to develop ART in canids results from marked differences between the reproductive physiology of dogs and other species. Dogs experience obligatory prolonged ovarian inactivity or anestrus [9], and as a result ovulation only occurs once or twice annually. Unlike in the human and mouse, canine oocytes are dark due to high lipid content, making it difficult to identify intracellular structures by light microscopy and to cryopreserve functional eggs and embryos [8]. Canid oocytes are also ovulated at an immature stage compared with other mammalian species (pre-germinal vesicle breakdown), and require 48–72 hrs in the oviduct post-ovulation to complete nuclear maturation [10].

Because of difficulty in obtaining oocytes after ovulation, previous attempts at IVF often used *in vitro* matured oocytes collected at various stages of the reproductive cycle, and often from ovarian follicles in various stages of development [11–15]. *In vitro* maturation, or IVM, generally has resulted in low rates of successful resumption of meiosis, with the exception being one study that found that use of oocytes from follicles larger than 2 mm yielded significantly higher rates of metaphase II development *in vitro* [16]. When paired with IVF, these previous attempts reported low rates of embryo production (2.2%-33.6%), only 1 blastocyst [14] and 3 morulae [11, 17, 18] out of hundreds of oocytes, and no live births. The field has been effectively constrained by not having a successful IVF protocol with which one could assess the fertilization competence of IVM-derived oocytes.

Successful IVF requires a) sperm that have acquired the ability to fertilize through a process known as “capacitation” [19], b) high quality, developmentally competent oocytes, c) optimal fertilization/embryo culture conditions, and d) an embryo transfer recipient with an oviductal/uterine environment that will support implantation and successful pregnancy. Lack of any one or several of these requirements would lead to failure; therefore, we designed our experimental approach around these factors.

An *in vitro* culture medium was previously developed for canine capacitation [20]. However, this medium omitted magnesium because its presence delayed the incidence of spontaneous acrosome exocytosis (AE). Magnesium is an important co-factor for glycolytic enzymes and has been shown to promote acrosome exocytosis (AE) via a Ca^2+^-Mg^2+^-ATPase in bull and ram spermatozoa [21]. Physiological AE is stimulated by progesterone (P4) and/or zona pellucida proteins [22]. Therefore, we evaluated the impact of Mg^2+^ on the ability of dog sperm to undergo physiologically relevant AE and hyperactivated motility, two hallmarks of capacitation. As canine oocytes rely so heavily on the oviductal environment to support maturation, we also tested whether additional time in the oviduct beyond attainment of nuclear maturation would be beneficial for fertilization. Finally, *in vivo*, circulating and local P4 levels begin to rise prior to the LH surge and subsequent ovulation [23]. This led us to test whether supplementation of P4 might enhance fertilization rates.

In the current study we determined that: 1) magnesium had positive effects supporting sperm hyperactivation and acrosome exocytosis, two functions essential for fertilization competence; 2) Day 6 post-LH surge was optimal for obtaining developmentally competent oocytes; 3) there was no significant effect of progesterone on embryo production; 4) male donor significantly influenced oocyte fertilization and embryo development in the dog; and 5) it is possible to produce live young from IVF-derived, frozen-thawed embryos transferred into the oviduct. Generation of a successful protocol for IVF in the dog lays the foundation for application of gene-editing technologies and also provides a means to perform gamete rescue in endangered canid species.

## Materials and Methods

### Animals and Estrus/Ovulation Monitoring

Thirty-three proven-breeder beagles (aged 1.5–5 years) and a single hound (age 1.5 years) from Marshall BioResources (North Rose, NY) were monitored during natural (unsynchronized) cycles from late anestrus through estrus. Animals were group housed on a 12 hr/12 hr light/dark cycle and fed once daily laboratory canine diet #5006 (LabDiet, St. Louis, MO) and provided water *ad libitum*. All experimental protocols were reviewed and approved by the Institutional Animal Care and Use Committee of Cornell University (protocol #2011–0004). Blood was drawn alternately from cephalic and saphenous veins 3–7 days a week, with daily sampling once proestrus was detected via either presence of serosanguinous discharge from the vulva and/or serum progesterone values higher than 0.4 μg/ml. Collected blood was allowed to clot, then centrifuged at 700g for 10 min to separate serum which was evaluated via chemiluminescent immunoassay (Immulite, Diagnostic Product Corporation, Los Angeles, CA), previously validated for use in the domestic dog [24] at the Animal Health Diagnostic Center at Cornell University. The days of the LH surge (Day 0) and ovulation (Day 2) were identified based on progesterone values of 1.5–2.5 and 4.5–5.5 ng/ml, respectively [24, 25]. Day 0 was confirmed using the Witness LH kit (Synbiotics, Kansas City, MO) when this product was available.

### Gamete/Embryo Collection and Handling

Canine capacitation media (CCM), was modified from Mahi and Yanigamachi [20]. CCM was supplemented with Hepes (25 mM) as a buffer and 0, 0.1 or 1 mM magnesium chloride. For incubating sperm under non-capacitating conditions, sodium bicarbonate and BSA were omitted.

Five proven-breeder male dogs (two Labrador retrievers, one beagle, and two cocker spaniels), aged 2–10 years, were used as semen donors. The first and second sperm-rich fractions were collected by manual stimulation following a minimum abstinence period of 2 days. The semen was centrifuged at 100g for 1 min to remove debris, then diluted in CCM to wash before a second centrifugation at 400g x 5 min to pellet the sperm. Sperm were re-suspended in modified CCM then incubated in polyvinyl alcohol-coated tubes at a concentration of 7.5 x 10^6^ sperm/ml at 38°C with 5% CO_2_ in air for capacitation. Sperm were incubated under non-capacitating and capacitating conditions for 4 hr and then assessed for motility, hyperactivated motility, and AE. To assess motility parameters, 25 μl sperm were pipetted under an elevated coverslip on a prewarmed slide and observed under a phase-contrast miscroscope (Optiphoto; Nikon, Tokyo, Japan). The percentage of progressively motile sperm and hyperactive sperm, as determined by the asymmetrical, “star-spin” pattern of motility in aqueous medium [26], were expressed as a percentage of total sperm counted (n > 200 sperm). AE was analyzed by incubating the sample with 10 μM progesterone for 1 hour, then processing for Coomassie assessment of AE as described previously [27]. AE was expressed as a percentage of total sperm counted (n > 200 sperm).

Spays were performed by Cornell Center for Animal Resources and Education veterinarians. On Days 5 or 6, dogs were spayed and entire tracts immediately transported to the laboratory in warmed PB1 [28, 29]. Oviducts were dissected out of the bursa and flushed with PB1 using a 23 G winged infusion set (Teruma Medical Products, Elkton, MD) connected to a 20 ml Leur lock Norm-ject syringe. Oocytes were washed and IVF and embryo culture were performed in NCSU [30] supplemented with 1% BME and 1% Minimum Essential Medium (MEM) Non-essential Amino Acids solution (Sigma) called cNCSU. Only high quality oocytes with homogeneously dark cytoplasm [16] and normal morphology (spherical, intact membrane) were utilized for the study. Oocytes observed to be degenerated or otherwise abnormal at time of collection or by the 24 hr mark were not included in analysis (n = 16). In most cases, the high lipid content and dense layer of cumulus cells surrounding post-ovulatory dog oocytes [10] obscured our ability to observe an extruded polar body. Oocytes were washed in medium before transfer to fresh, pre-equilibrated 100 μl droplets of the various media overlayed with mineral oil for IVF. *In vitro* capacitated sperm (incubated for 2.5–3.5 hrs under capacitating conditions) were added to the oocytes in the 100 μl IVF droplet at a final concentration of 1 million sperm / ml. The gametes were co-incubated for 14 hr at 38°C and 5% CO_2_, 5% O_2_ and 90% N_2_ in a humidified hypoxia chamber.

After 14 hr, presumptive zygotes were washed by gentle pipetting in fresh medium to remove loosely attached sperm and/or cumulus cells, then transferred to individual, 50 μl droplets, pre-equilibrated in mineral oil. Images were taken with Spot Software 4.1 (Diagnostic Instruments, Sterling Heights, MI). Presumptive zygotes were returned to culture under the same temperature and gas conditions. Embryo cleavage was evaluated at 48 hr post-IVF, then embryos were either cryopreserved or medium exchanged for further culture, re-evaluating cleavage stage every 24 hr (e.g. 72, 96 hr).

Embryos were vitrified and thawed using the commercially available Vit Kit Freeze and Thaw kit (Irvine Scientific, Santa Ana, CA) according to manufacturer instructions. Cryopreserved embryos were allowed to recover in Medium 199 supplemented with 20% fetal bovine serum (v/v) for 2–3 hr prior to transfer into five recipient beagles experiencing natural estrous cycles on the appropriately synchronized day (Day 8 or 10). Embryos were transferred in groups based on progesterone treatment.

For Transfers #1–3, embryos were frozen between 48–55 hr post-IVF (Day 8 post-LH surge, embryos at the 2–4 cell stage) and transferred into the cranial uterine horn. Embryos frozen between 94–98 hr post-IVF (Day 10, embryos at the 4–8 cell stage) were also transferred to the uterine horn in Transfer #4. In designing this transfer, we hypothesized that there might be some undescribed effect of exposure to seminal plasma and/or maternal recognition of pregnancy that caused failure of the first three transfers. We therefore performed AI with 1 ml semen from a 3 yr old Labrador donor on Day 3 prior to the transfer on Day 10. For uterine horn transfer procedures, a stab incision was made into the cranial portion of the uterine horn, exposed via laparotomy, and embryos in minimal medium (Medium 199 + 20% FBS, 15–40 μl) were gently pipetted into the lumen using a large-orifice 200 μl pipette tip. In Transfer #5, Day 8 frozen-thawed embryos were transferred into the oviduct of the recipient. For oviductal transfer, both left and right ovarian bursas of a single recipient hound experiencing a natural estrous cycle were exteriorized via laparotomy. The suspensory ligament was manually dissected and a slit was made through the bursal window to better access the infundibulum. Embryos were drawn up into a 3.5 FR, 4.5” (11.4 cm) tom cat catheter (Argyle, Covidien, Mansfield, MA) with a 1 cc syringe in 20 μl medium, then the catheter was advanced approximately 2.5 cm into the oviduct via the infundibulum and the embryos were slowly dispensed. Pregnancy diagnosis and checks were performed via ultrasound weekly beginning Day 29 post LH-surge, and all births were via planned Caesarian section on Day 65.

### Experiments and Statistics

#### Collection Day Evaluation

Oocytes were flushed from excised oviducts on Day 5 (N = 7, n = 37) and Day 6 (N = 15, n = 44) with the day of LH surge counted as “Day 0” by convention) and fertilized in the absence of progesterone. Post IVF, presumptive zygotes were transferred to embryo culture media as described above and assessed daily for cleavage and embryo stage.

#### Effect of Progesterone

Day 6 oocytes (n = 146) from 24 dogs were collected and IVF and embryo culture. Initially, half the oocytes collected from each individual were exposed to 6 μg/ml progesterone (in DMSO, Sigma) added to the cNCSU for both IVF and embryo culture (N = 15, n_total_ = 90). In the last 9 dogs, all oocytes (n = 56) were cultured with P4. Embryos produced in this study were either vitrified for later embryo transfer into one of five recipient dogs, transferred “fresh” without freezing, or cultured until first appearance of degeneration.

#### Statistics

Differences between sperm capacitation metrics were evaluated by Student’s T test. Chi square / likelihood ratio test were used to determine differences between variables (Collection day, P4 supplementation, male sperm donor) on embryo development rates using JMP 10.0 Software (SAS, Cary, NC).

## Results

### Effect of Magnesium Supplementation on Sperm Capacitation

**Fig 1:**
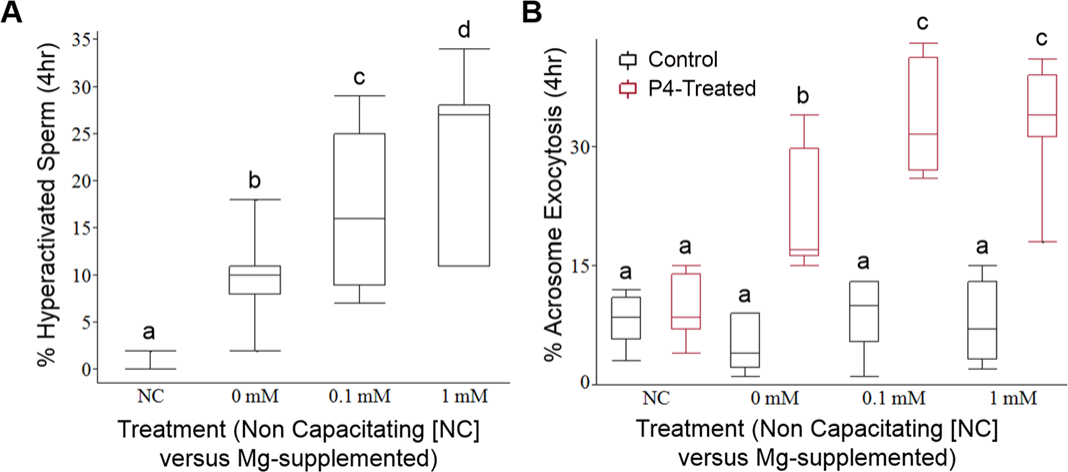
Effects of Mg^2+^ supplementation on hyperactivated motility and ability to undergo P4-stimulated AE. (**A**) Percentage of hyperactivated sperm after capacitation for 4 hr (N = 4 males, n_total_ = 3825 sperm) (**B**) Percentage of sperm undergoing AE spontaneously and in response to 10 μM P4 in each of the treatments after capacitation for 4 hr (N = 4 males, n_total_ = 7202 sperm). Letters indicate significant differences (P < 0.05) among treatment groups.

We first evaluated the effect of Mg^2+^ supplementation to canine capacitation medium (CCM [20]) on sperm motility, hyperactivation and ability to undergo AE in response to 10 μM P4. Progressive motility was unaffected by Mg^2+^ throughout the capacitation incubation. The percentage of sperm exhibiting hyperactivated motility increased in all conditions, but after 4 hr of incubation the percentage of sperm exhibiting hyperactivated motility was significantly higher (P < 0.05) in the 0.1 and 1.0 mM MgCl_2_ treatments than in the treatment without Mg or the non-capacitating control (Fig 1A). Moreover, a significantly higher percentage of sperm (P < 0.05) in the 0.1 and 1.0 mM MgCl_2_ treatments exhibited a P4-induced AE response than in the 0 mM MgCl_2_ treatment or non-capacitating control at the 4 hr time point (Fig 1B). However, addition of Mg^2+^ had no effect on spontaneous AE (as measured in the absence of P4). There was no difference in the AE response at 4 hr between the 0.1 mM and 1.0 mM MgCl_2_, treatments (Fig 1B). Our data showed that hyperactivation and rates of P4-induced AE were significantly improved in the presence of Mg^2+^, leading us to use CCM with 1.0 mM MgCl_2_ for our IVF experiments.

### Assessment of Collection Day and Progesterone on Embryo Development

**Fig 2:**
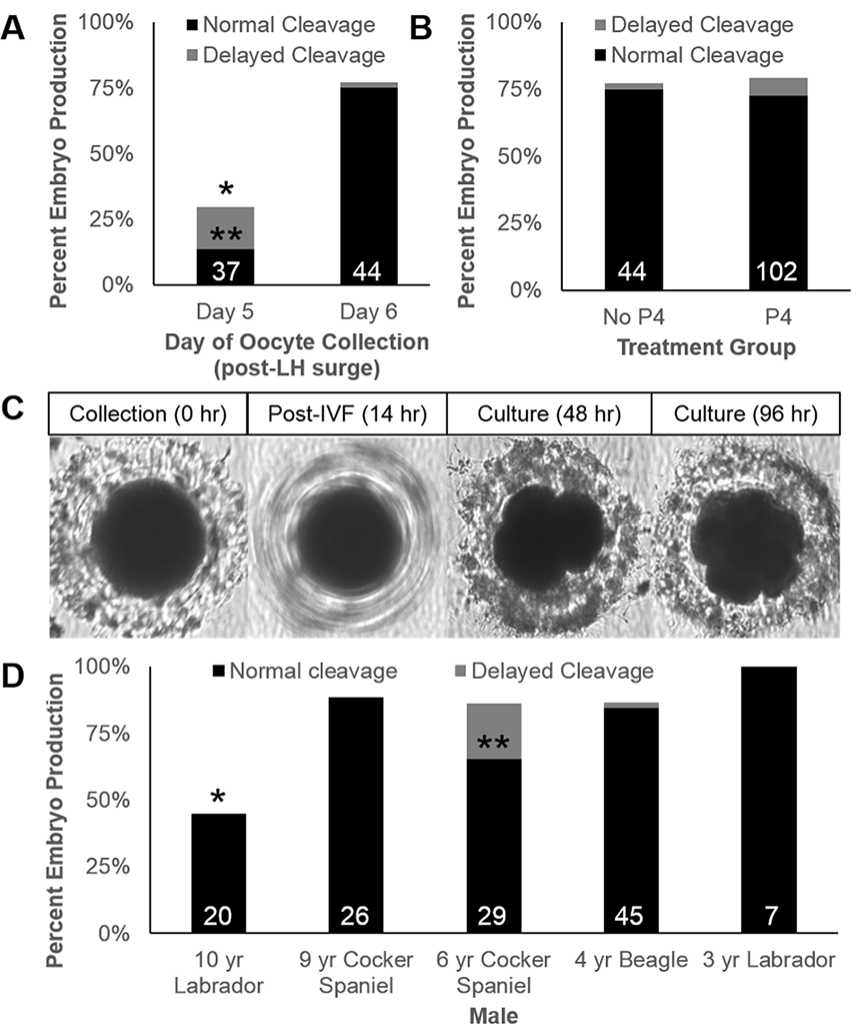
Development of embryos from Day 6 oocytes fertilized *in vitro* with sperm capacitated with Mg^2+^. **Normal cleavage = black bars, delayed cleavage = grey bars**. (**A**) Proportion of oocytes collected on Day 5 versus Day 6 developing into embryos. Asterisks (*,**) indicate significant differences for day for total and delayed cleavage percentages, respectively, (**B**) Proportion of oocytes developing into embryos in unsupplemented versus P4-supplemented cNCSU, (**C**) An individual oocyte at collection though fertilization developing into an 8-cell embryo. A focused image of the presumptive zygote (14 hr mark) was not possible due to motility of attached sperm (**D**) Effect of male sperm donor on cleavage rates. Asterisks (*, **) indicate significant differences for that male in total and delayed cleavage, respectively.

Next, sperm capacitated with in the presence of magnesium were used for *in vitro* fertilization of oocytes collected on Day 5 or 6 post-LH surge, allowing us to compare the effect of the duration of oviductal maturation. From Day 5 oocytes (N = 7 dogs), a total of 11 embryos were produced from 37 oocytes (29.7% embryo cleavage). However, over half of those that cleaved experienced delayed cleavage (n = 6, or 18.9% of oocytes), defined by embryo production occurring more than 48 hrs post-IVF (Fig 2A). Conversely, for Day 6 oocytes (N = 15 dogs), 34 of 44 oocytes cleaved (77.3%, P < 0.05), with only one of the 44 (2.3% of total oocytes) experiencing delayed cleavage (P < 0.05, Fig 2A).

Oocytes collected on Day 6 and fertilized in the absence of progesterone were then compared with those fertilized in the presence of progesterone (N = 24, n = 102). No significant effect (P > 0.05) of P4 supplementation on embryo cleavage was observed Fig 2B. For oocytes cultured in the absence of P4, 34 of 44 (77.3%) successfully fertilized and developed, whereas 81 of 102 (79.4%) did the same in the presence of P4 (Fig 2B, P > 0.05). A small percentage of the total had delayed cleavage (n = 8 embryos; 5.5% of the total). Embryos which cleaved generally were at the 4-cell stage after 48 hr culture, few oocytes experienced initial cleavage (2 cell stage) beyond the 48 hr mark in either treatment, and the impact of P4 was not statistically significant (Fig 2B). Only a portion of normally-cleaving embryos (N = 6, n = 21) were cultured to the 96 hr mark, or Day 10. In this group, rates of development to the 8-cell stage were high (15 of 21, or 71.4% of normally cleaving embryos; 15 of 34 or 44.1% of total oocytes cultured for 96 hr); however, these embryos did not develop beyond the 16-cell stage. Images of an individual oocyte taken at collection, during IVF (image not focused due to the motility of attached sperm causing the oocyte/presumptive zygote to ‘spin’), and after 96 hr culture are shown in Fig 2C.

Post-hoc analysis revealed an effect of one donor male on total fertilization success and another male for delayed-cleavage of embryos (Fig 2D–data exclude trials performed with multiple sperm donors). While there were no visible abnormalities or obvious capacitation differences in the sperm from the 10 year-old Labrador, only 9 of 20 oocytes inseminated with his sperm cleaved (45%). If one excludes oocytes incubated with this sperm from calculations, the rate of embryo development increased (total of 84% for unsupplemented, and 88.8% for P4-supplemented), but there was still no statistically significant difference between treatment groups for normally cleaving embryos. Higher rates of delayed cleavage in oocytes fertilized by sperm from a 6 year-old cocker spaniel were observed and represent a second male effect.

### Transfer of IVF-derived embryos

**Fig 3:**
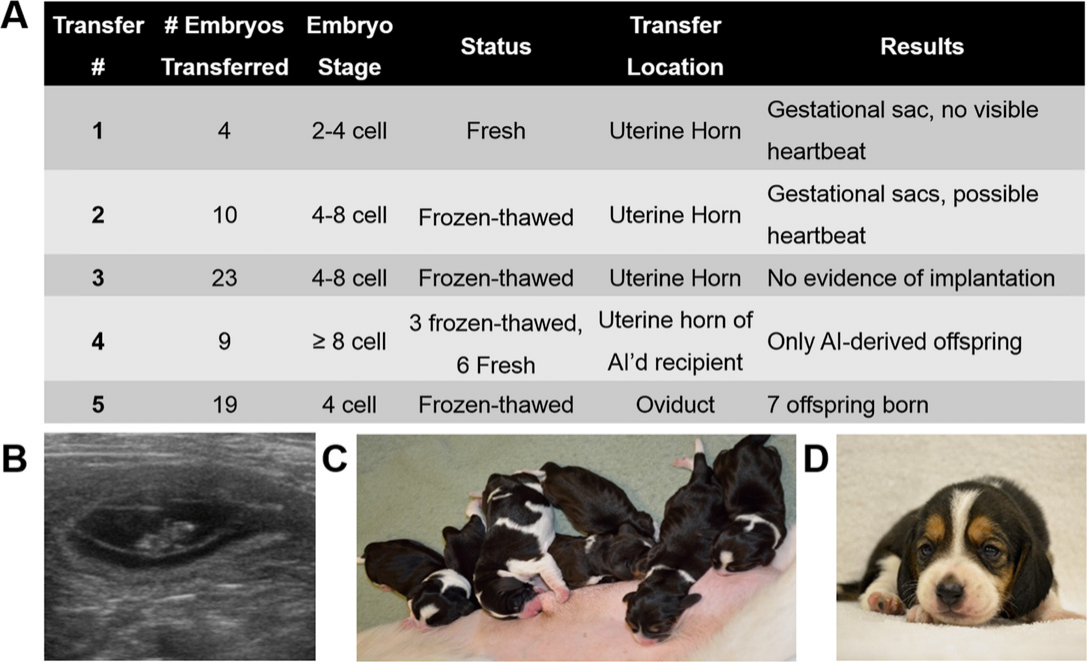
Results of transfer of IVF embryos. (**A**) Embryo stage, status, transfer location and the results of IVF-derived embryo transfers. (**B**) Ultrasound image of a normally-developing embryo imaged Day 29 from Transfer #5. (**C**) 7 healthy puppies were born by planned Caesarian section. (**D**) Normally developing beagle puppy at 3 weeks of age.

To assess the developmental potential of embryos produced in our system, we transferred them to naturally synchronized recipients. A previous study in the dog demonstrated the capacity of early stage *in vivo* derived embryos (2- to 8-cell) to develop to term after transfer into the uterine horn [31], although *in vivo* embryos do not reach the uterine horns until they are morulae on approximately Day 11 [32]. On this basis, we performed transfers #1–4 into the proximal uterine horn. In the fourth transfer, we also artificially inseminated the bitch using sperm from a male not utilized for any of the IVF, in case some signal produced by early embryos was necessary for later development, influencing either the uterine environment or producing beneficial paracrine effects on the transferred embryos. No birth of pups from IVF-derived embryos resulted from any of these transfers, although early fetal development was observed in three recipients via ultrasound (Fig 3A).

**Fig 4:**
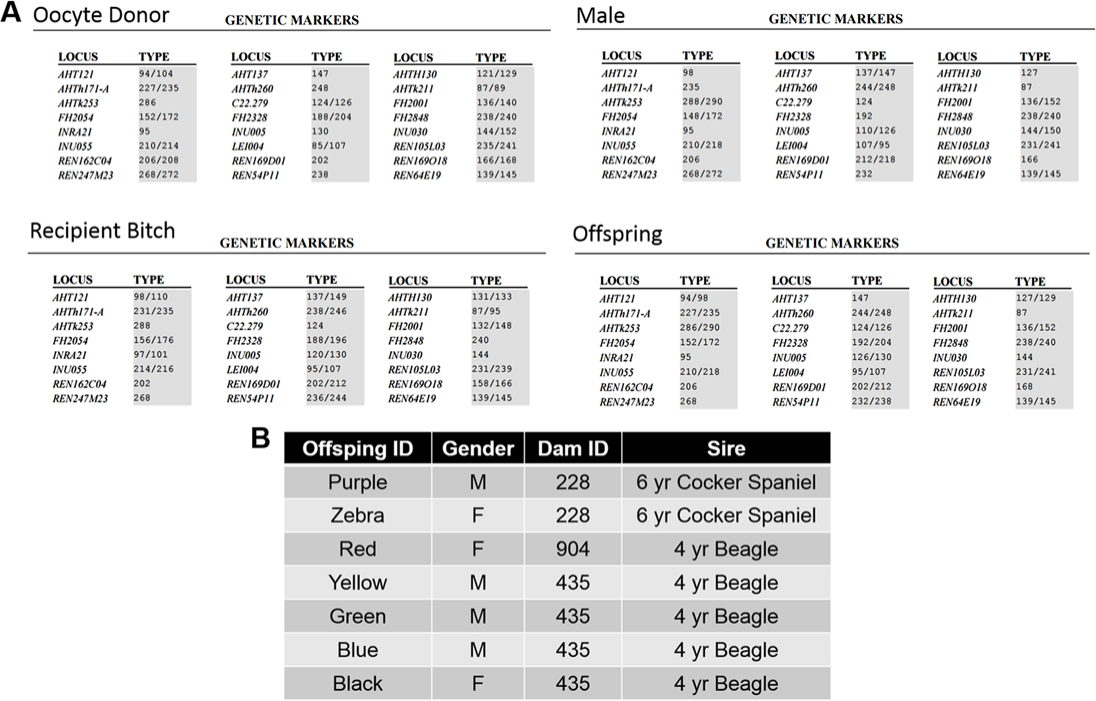
Parentage Results. (**A**) Representative parentage results via Veterinary Genomics Laboratory with genetic parents (oocyte and sperm donor male), recipient bitch, and offspring. The recipient did not qualify as a parent of any offspring. (**B**) Summary table of parentage of the seven offspring.

In Transfer #5, 19 embryos were transferred into the lumen of the oviduct via cannulating the infundibulum. Seven gestational sacs were visible ultrasonographically on Day 29 (Fig 3B), resulting in seven live offspring (3 females, 4 males) born by planned Caesarian section on Day 65 (Fig 3C). All pups exhibited normal behaviors and growth through weaning and the first three months of life (Fig 3D). Parentage of offspring (ruling out the recipient as mother, Fig 4A) was confirmed independently through DNA samples submitted to the Veterinary Genetics Laboratory (UC Davis, Davis, CA) and Animal Genetics (Tallahassee, FL). Results determined three oocyte donors and two sperm donors/sires from three IVF procedures were represented in the litter (Fig 4B).

## Discussion

Attempts at IVF in the dog previously resulted in very low rates of embryo development and no live births. We initially hypothesized that this lack of success was due to prior use of *in vitro* matured oocytes of unknown developmental competence. Here, utilization of an improved sperm capacitation protocol and Day 6 *in vivo* matured oocytes enabled us to consistently produce embryos with high efficiency.

Although studies of nuclear status have previously determined that canine oocytes resume meiosis and reach metaphase II by 72 hrs post ovulation (Day 5) [10], *in vivo*-matured oocytes collected at this stage performed relatively poorly in our IVF system. Dogs exhibit a long window in which oocytes can be fertilized; for example, intrauterine AI has produced pregnancies up to 10 days after the LH surge [33]. Together, these data suggest additional steps beyond nuclear maturation are needed for fertilization. We did not detect any factor that influenced development up to the 8-cell stage. Our data suggest that the main obstacles previously encountered were the result of some combination of sperm function and oocyte maturation.

Surprisingly, P4 supplementation had no beneficial effect on embryo cleavage. Both the ovarian follicle [34] and the oviduct [35, 36], display increasing levels of P4 receptor during the perivovulatory period in the dog. Recent evaluations of the composition of canine oviductal fluid demonstrated high levels of P4 [37]. This led us to hypothesize that progesterone has a role in oocyte maturation through early embryo development. Although no effect of P4 on early embryo development was observed, our data do not rule out whether this hormone plays one or more functions in the periovulatory canine oviduct, on the immature oocyte, the oviductal epithelium, and/or the sperm. For example, exogenous P4 can affect Ca^2+^ influx, capacitation and acrosome exocytosis in murine sperm [38]. That being said, we saw no evidence for an effect of P4 to promote fertilization of Day 6 oocytes.

Several studies in the domestic dog have noted a relatively wide distribution of early embryonic stages in the oviduct post-fertilization [10, 32, 39], suggesting that variation or ‘delay’ in cleavage time was not necessarily a product of the *in vitro* culture conditions. Consistent with this, we observed that in some cases the late-cleaving oocytes ‘caught-up’ developmentally with the other embryos in their cohort. Difficulty overcoming the 16-cell stage has been reported in other studies of canine IVF [13], and when culturing *in vivo*-derived dog embryos [40]. Together, the data suggest an *in vitro* developmental block just beyond the point of embryonic genome activation (8-cell stage in the dog [41]). To circumvent this, we transferred embryos at the 4-cell stage. Success in terms of both implantation [42] and live birth [31] has been shown in the transfer of early stage embryos into the uterine horn although embryos do not reach this portion of the reproductive tract until Day 11 post LH surge (~16-cell stage) *in vivo* [32]. However, transfer of IVF-derived early stage embryos into the uterine horn did not result in any live births in this study.

Reported success at embryo transfer ranges from 51.9% (using fresh *in vivo*-derived 8-cell to blastocyst-staged embryos; [43]) to 9.1% (fresh embryos cryopreserved in Cryotops [29]). Our success rate of 36.8% was obtained using 4-cell stage, IVF-derived embryos cryopreserved with a “VitKit,” thawed and transferred into the oviduct. Direct comparisons using human embryos between the ‘open’ Cryotop and ‘closed’ VitKit cryopreservation systems determined no significant difference in blastocyst survival or pregnancy outcome [44]. Although early stage dog embryos have been transferred successfully into the uterine horn [29, 31], our intrauterine transfers of 2–8 cell embryos cryopreserved with the VitKit failed. This suggests that either difference in site of embryo transfer was more important than method of cryopreservation, or that the optimal site of embryo transfer might depend to some extent on the method of freezing.

From a practical perspective, our high success rate holds promise for the more widespread use of embryo cryopreservation in the dog. Because females only cycle once or twice a year, cryopreservation of embryos would allow long-term storage until another female reached the appropriate stage of cycle to function as a recipient. The ability to cryopreserve embryos also allows movement of valuable genetics across distances, which will benefit gene flow among isolated or fragmented conditions.

In the present study, supplementation of the standard CCM with Mg^2+^ yielded significant improvements in hyperactivated motility and ability to undergo physiologically-induced AE, sperm functions required for fertilization. Oocytes collected on Day 6 post-LH surge fertilized readily in both minimally-supplemented and more complex media, with no influence of progesterone addition. Four-cell embryos fertilized in this IVF system and cryopreserved using VitKit prior to transfer into the oviduct resulted in the live birth of seven puppies.

Successful IVF makes possible a variety of applications, including opening new opportunities for gamete rescue of endangered species or targeted propagation of domestic dogs of high genetic value. Importantly, the dog is also a preferred model for studies in stem cell transplantation and gene therapy, and has well-characterized breed predispositions to hundreds of traits and pathologies that also plague humans. But the full potential of dog genetics has not been realized because of lack of IVF/ART. Methods shown here enable new gene-editing technologies such as CRISPR/Cas to be applied to the dog in an efficient manner. This approach will allow genes identified as candidates to cause or predispose an individual to a pathology or undesired trait to be tested empirically. This in turn will facilitate both development of targeted treatments and genetic screening tests to be used to remove those genes from affected breeds, with broad positive impacts on human and companion animal health and welfare.
